# Prevalence and Factors Associated with Sexually Transmitted Infections among HIV Positive Women Opting for Intrauterine Contraception

**DOI:** 10.1371/journal.pone.0122400

**Published:** 2015-04-10

**Authors:** Othman Kakaire, Josaphat Kayogoza Byamugisha, Nazarius Mbona Tumwesigye, Kristina Gamzell-Danielsson

**Affiliations:** 1 Department of Obstetrics and Gynecology, Makerere University College of Health Sciences, Mulago Hospital Complex, Old Mulago Hill, Kampala, Uganda; 2 School of Public Health, Makerere University College of Health Sciences, Mulago Hospital Complex, Old Mulago Hill, Kampala Uganda; 3 Department of Women’s and Children’s Health, Division of Obstetrics and Gynecology, Karolinska Institutet, Karolinska University Hospital, Stockholm, Sweden; Leibniz Institute for Prevention Research and Epidemiology (BIPS), GERMANY

## Abstract

**Background:**

Women living with HIV/AIDS (WLHA) are a high risk group for sexually transmitted infections (STIs). However, the majority of women with STIs are asymptomatic. Data on prevalence of STIs among WLHA in Uganda are limited. The objective of the study was to determine prevalence and factors associated with STIs among WLHA opting for intrauterine contraceptive device (IUD).

**Methods:**

Three hundred fifty one WLHA deemed free of STIs using a syndromic logarithm were enrolled into the study. Endo-cervical swabs were taken before IUD insertion and PCR test for Nisseria gonorrhea (NG), Trichomonas vaginalis (TV) and Chlamydia trachomatis (CT) infections conducted.

**Results:**

Participants’ mean age was 29.4 ± 6.2 years, 83% were under 35years, 50% had secondary education and 73% were married. The majority (69%) had disclosed their HIV sero status to their spouses, 82% used Cotrimoxazole prophylaxis, 70% were on antiretroviral therapy, 90% had CD4 count greater than 350, about 60% reported condoms use and 70% were of parity 2-4. Over 50% of the participants’ spouses were older than 35 years and 72% had attained secondary education. STIs prevalence was 11.1%, (95% CI 7.8-14.4) and individual prevalence for TV, NG, and CT was 5.9%, 5.4% and 0.9% respectively. Factors independently associated with STI were having primary or less education (OR= 2.3, 95% CI: 1.09 - 4.85) having a spouse of primary or less education (OR= 3.3, 95% CI: 1.6 - 6.78) and muslim faith (OR= 0.2, 95% CI: 0.04 - 0.78).

**Conclusion:**

STI prevalence was 11.1%. TV and NG were the commonest STIs in this population. Having primary or less education for both participant and spouse was associated with increased risk while being of muslim faith was associated with reduced risk of STI.

## Introduction

Sexually transmitted infections (STIs) remain a global public health challenge. The latest World Health Organization estimates indicate that 498.9 million new STIs occur annually among people aged between 15 and 49 years [1]. Sub Saharan Africa has an STI incidence of 240/1000 which is the highest in the world [1]. It is estimated that 8.3, 21.1 and 59.7 million new cases of Chlamydia trachomatis (CT), Neisseria gonorrhea (NG) and Trichomonas vaginalis (TV) infections take place in sub Saharan Africa annually [1]. Evidence shows that in African settings, NG, CT. and TV increases HIV virus acquisition and transmission [2–7]. The inflammatory process resulting from STIs increases viral shedding of HIV-1 in the genital tract [8–10], increasing the risk of HIV-1 transmission to the sex partners.

Accurate national data on the prevalence of individual STIs is not available in Uganda but epidemiological studies show that STIs are a public health problem. Among female adolescents, the prevalence of CT, NG and TV has been reported to be 4.5%, 9% and 8% respectively [11]. Whereas among a cohort of women involved in high risk sexual behavior the prevalence of CT, NG and TV were 9%, 13% and 17% respectively [12]. In Uganda, like many low to middle income countries (LMICs), the syndromic management for STIs is being implemented. Though, syndromic management is a cost-effective strategy to treat symptomatic STIs, its value in the detection and management of asymptomatic STIs is limited [13]. Evidence shows that 30–80% of the women with NG, 85% with CT and 80% with TV are asymptomatic [14–17]. Hence a large number of women are living with asymptomatic STIs.

Untreated STIs among women receiving intrauterine contraception can be problematic. Placement of intrauterine contraception can transmit STI microorganisms in asymptomatic women into the uterine cavity leading to development of pelvic inflammatory disease (PID) [18]. PID associated with intrauterine contraception is commonest in the first 20days of intrauterine contraception initiation [18,19]. Furthermore, studies show that PID related to intrauterine contraception depends on background STI prevalence in a given population [19]. WLHA are a high risk group for STIs [20] with increased risk of acquiring cervical CT and NG infections [21]. The United States Centre for Disease Control and Prevention (CDC) recommends women at high risk of STIs to undergo laboratory test before placement of intrauterine contraceptive devices (IUD) [22]. However, in LMICs such services are not readily available. The aim of this study was to determine prevalence and factors associated with asymptomatic STI among WLHA undergoing syndromic screening for STI prior to placement of the IUD.

## Methods

### Setting

The study was conducted in Mulago National Referral hospital, a public medical facility in Kampala, Uganda. It is also the University teaching hospital for Makerere University. The hospital has several departments but work for this study was carried out in the Makerere University Joint Aids Program (MJAP) and the family planning clinics. The MJAP is an AIDS patient care facility with an annual attendance of about 20,000 clients, 60% of whom are women of reproductive age. It offers services such as voluntary counseling and testing for HIV, management of opportunistic infections, antiretroviral therapy, contraceptive counseling, prevention services on mother to child transmission of HIV and supply of condom for the dual prevention of STI/HIV transmission and unwanted pregnancy. Response to HIV/AIDS care is monitored using quarterly CD4 cell counts. Patients whose CD4 cell count reduce or fail to increase get HIV viral load estimated.

The MJAP and family planning clinics are 10 meters apart. The family planning clinic offers a number of services including; family planning counseling and follow-up, syndromic management of STIs, voluntary counseling and testing for HIV, cervical cancer screening and family planning service provision. There is a wide range of contraceptive methods ranging from the short term hormonal methods to long term and permanent methods. Annually 30,000 clients are seen at the clinic and about 3,000 women opt for intrauterine contraception. Eight midwives with over 10 years’ experience of contraceptive service provision including intrauterine contraception insertion and removal run the clinics.

### Study Population

We report findings from the baseline survey of an ongoing clinical trial, “safety and acceptability of levonorgestrel intrauterine system and copper intrauterine device (SALIC)” registration number PACTR201308000561212, which is investigating the safety and acceptability of intrauterine contraception among women undergoing HIV/AIDS care at Mulago hospital. Women were eligible for the parent trial if they were attending HIV/AIDS care at Makerere Joint Aids Program clinic, were 18 to 49years old, had a stable sexual partner and wanted to avoid pregnancy for at least one year. Women with menstrual irregularities, uterine abnormalities, severe dysmenorrhea, AIDS but not on antiretroviral therapy and those who had a history of STIs currently or in the three months preceding enrollment were excluded. Women who reported their husbands to have multiple extramarital partners or a recent history of STI were also excluded.

### Measurement

From September to December 2013, the research team approached women as they awaited HIV/AIDS care at the MJAP clinic. The women were educated about family planning and the study introduced to them. One thousand five hundred women interested in the study were referred to the family planning clinic where method specific counseling was done. Nine hundred women opted for intrauterine contraception and were screened for STIs using the syndromic logarithm. Seven hundred and three women were deemed free of STI after syndromic screening. The purpose of the study was explained to the women and those who consented to participate in the study were interviewed using a pretested questionnaire.

The questionnaire collected information on demographic, reproductive and sexual history including; history of suffering or being treated for sexually transmitted infections in the past three months, number of sexual partners in past twelve months, menstrual flow pattern, Cotrimoxazole use and antiretroviral therapy. After the interview, women who were at low risk of STI and opted for intrauterine contraception had a physical examination including a pelvic examination. The women who did not have abdominal and cervical motion or adnexal tenderness, purulent vaginal or cervical discharge, and inflamed cervix were deemed fit for insertion of an intrauterine contraceptive.

Before intrauterine contraceptive (IUD) was inserted, the 703 women were randomly assigned to have the IUD inserted right away or to have a laboratory test for STIs before insertion. The 351 women randomized to the laboratory arm had an endo cervical swab taken from the cervix. A sterile cotton swab was inserted in the cervix, rotated clock wise through 360° and kept in a transport medium at room temperature until analysis. Samples were analyzed daily within 4 hours of sample collection.

DNA was extracted from high vaginal swab materials using master pure DNA extraction kit, (Epicentre Biotechnologies), as per the manufacturer’s Instructions. Following DNA extraction, real time validated in house PCR assay was done for detection of NG, CT and TV in triplex format. It was done using primers and labeled probes which target NG porA pseudo, CT orf8, TV 18sRNA and human β-actin gene as an internal control using the quant fast multiplex kit (Qiagen, www.qiagen.com) using 5plex rotor gene Q600 plat form. The probes and primerswere designed using primer blast tool (http://www.ncbi.nlm.nih.gov/tools/primerblast/primerinfo.html) and synthesized by Eurofins MWG Operons. For each organism, two primers and one labeled probe were used. To detect NG, we targeted porA gene, forward primer 5’- ccggaactggtttcatct-3’, reverse primer 5’gttttagcggcagcattc -3, probe sequence 5’ Cgtgaaagtagcaggcgtataggcggactt -3’ and probe label 5’-FAM, 3’-BHQ1 were used. While the detection of CT involved targeting the orf8 gene using forward primer 5’-taggcgtttgtactccgtc -3’, reverse primer 5’- atgcactttctacaagagtacat -3, probe sequence 5’ Tgcagcttgtagtcctgcttgaga -3’ and probe label 5’- ROX 3’-BHQ2. TV was detected by targeting 18s RNA gene using forward primer 5’- ggaaacttaccaggaccaga-3’ reverse primer 5’- aaggacttcggcaaagtaaat-3’ probe sequence Tgcatggccgttggtgg and probe label 5’-JOE, 3’-BHQ. For internal control, human β-actin gene was targeted using forward primers 5’- cactgtgcccatctacg-3’ reverse primer 5’- gttaatgtcacgcacgatttc-3’ probe sequence 5’ Gatccttcaccgagcgcg-3’ and probe label 5’-HEX 3’-BHQ1. The reaction was done in a volume of 25μl containing probes and primer to a final concentration of 0.2μm and 0.5μm respectively, 12.5 μl of 2xquantifast multiplex PCR master mix, 5.5μl of PCR grade water and 5 μl of DNA sample. PCR water and DNA extracted from ATCC Strains (NG ATCC49226, CT ATCC VR-879D and TV ATCC3001D) were used as negative and positive controls. The amplification was done using the following program; initial denaturation/ enzyme activation step for 5min at 95°c, followed by 40 cycle of denaturation at 95°c for 45s, annealing at 53°C for 45s and extension at 60°c for 45s. Acquisition was done during extension on the green, orange and red. Sample with a typical sigmoid curve and critical cycle value of ≥35 for human β-actin and any of target pathogen was taken as positive.

For quality control and quality assurance, DNA extraction and PCR amplification steps weredone in separate rooms which didn’t communicate to each other. Sterile nuclease/DNAmaterials including aerosol resistant barrier tips were used throughout the process. Negative amplification, negative template control and post amplification controls were run for every batch and PCR inhibition Control was run for every Sample to rule out failed PCR.

### Statistical analysis strategy

The analyses include the baseline data for the 351 participants who underwent laboratory screening for STIs prior to insertion of intrauterine contraception. Data were double entered in EpiData version 3.1 soft ware, cleaned and exported to Stata 12 soft ware for analysis. All samples that tested positive for CT or NG or TV were considered positive for STI. Participants were considered as having an STI if NG or CT or TV was detected by PCR in the endo-cervical sample collected. Descriptive statistics (minimum, maximum, mean, and standard deviation) were applied for continuous variables and simple percentages for categorical variables. Univariate analysis was conducted to describe background characteristics of the study participants (Table 1). The prevalence of STIs was calculated as proportions of participants with positive PCR test for NG or CT or TV. Individual prevalence for NG, CT, and TV were also calculated as proportions (Table 2). Chi-square or Fisher’s exact t- tests were performed, as appropriate, to analyze the bivariate relationships between the factors of interest and the outcome defined as being PCR positive for either GC or TV or CT infections. Variables with a p value of ≤0.2 at bivariate analysis and biologically important variables were selected for multivariable logistic regression (Table 3) to determine factors which were independently associated with a positive PCR test for sexually transmitted infections.

**Table 1:** Background characteristics of the 351women living with HIV opting for intrauterine contraception at Mulago Hospital.

Characteristics		STI Negative n = 312 (%)	STI positive n = 39 (%)
Age (completed years)			
	≤35years	255 (81.7%)	36 (92.3)
	>35 years	57 (18.3%)	3(8.7)
Education Level			
	≥Secondary	17(56.1)	13 (33.3)
	≤ Primary	137 (43.9)	26 (66.7)
Religion			
	Catholic	110 (35.3)	16 (41.0)
	Protestant	98 (31.4)	17 (42.6)
	Muslim	68 (21.8)	2 (5.1)
	Born again	29 (9.3)	4 (10.3)
	Adventists	7 (2.2)	0
Marital status			
	Married	227 (72.8)	27 (69.2)
	Single	85 (27.2)	12 (30.8)
Participant alcohol use			
	Yes	54 (17.3)	9 (23.1)
	No	258 (82.7)	30 (76.9)
Spouse Education			
	≥ Secondary	231 (74.0)	20 (51.3)
	≤ Primary	81 (26.0)	19 (48.7)
Spouse Age			
	≤35years	154 (49.4)	25 (64.1)
	>35 years	158 (50.6)	14 (35.9)
Spouse alcohol use			
	Yes	116 (37.2)	19 (48.7)
	No	196 (62.8)	20 (51.3)
Disclosure of HIV status to partner			
	Yes	91 (70.2)	23 (59.0)
	No	93 (29.8)	16 (41.0)
Cotrimoxazole use			
	Yes	256 (81.1)	36 (92.3)
	No	59 (18.9)	3 (7.7)
ARV			
	Yes	273 (87.5)	37 (82.05)
	No	39 (12.5)	7 (18.0)
Condom use			
	Yes	178 (57.1)	19 (48.7)
	No	134 (43.9)	20 (51.3)
Cd4 count			
	≤350	72 (23.1)	13 (33.3)
	>350	240 (76.9)	26 (76.7)
Parity			
	Para 1	54 (17.3)	6 (15.4)
	Para2-4	208 (66.7)	28 (71.8)
	Para >4	50 (16.0)	5 (12.8)
Number of sexual partners in past one year			
	1	327 (88.1)	31 (79.5)
	≥2	37 (11.9)	8 (20.5)

STI = Sexually transmitted infections

**Table 2:** Distribution pattern of sexually transmitted infections.

Detected STI	Frequency n = 39	Percentage
TV only	18	46.2
NG only	15	38.5
CT only	2	5.1
NG + CT	1	2.5
NG + TV	3	7.7

TV = Trichomonas vaginalis, CT = Chlamydia trachomatis, NG = Neisseria gonorrhea

**Table 3:** Factors associated with STIs among women living with HIV adjusted for all other variables in the model.

Characteristics		Frequency N = 351	STI positive (%)	Crude OR/ 95%CI	P—value	Adjusted OR/ 95%CI	P—value
Age (completed years)							
	≤35years	291	36 (12.4)	1			
	>35 years	60	3(5.0)	0.4 (0.11–1.25)	0.111	0.5 (0.12–1.78)	0.26
Education Level							
	≥Secondary	188	13(6.9)	1			
	≤ Primary	163	26 (16.1)	2.6 (1.27–5.16)	0.009	2.5 (1.16–5.39)	0.019
Religion							
	Non Muslim	281	37 (32.2)	1			
	Muslim	70	2 (1.6)	0.2 (0.05–0.83)	0.026	0.2 (0.36–0.77)	0.023
Marital status							
	Married	254	27 (10.6)	1			
	Single	97	12 (12.4)	1.2 (0.58–2.45)	0.643	-	-
Participant alcohol use							
	Yes	63	9 (23.1)	1			
	No	288	30 (76.9)	0.70 (0.31–1.55)	0.378	1.0 (0.37–2.46)	0.929
Spouse Education							
	≥ Secondary	251	20 (8.0)	1			
	≤ Primary	100	19 (19.0)	2.71 (1.38–5.33)	0.004	3.1 (1.45–6.52)	0.004
Spouse Age							
	≤35years	179	25 (14.0)	1			
	>35 years	172	14 (8.1)	1.8(0.92–3.66)	0.086	0.5 (0.25–1.19)	0.125
Spouse alcohol use							
	Yes	135	19 (14.1)	1			
	No	216	20 (4.8)	0.6 (0.32–1.22)	0.165	0.7 (0.33–1.53)	0.379
Disclosure of HIV status to partner							
	Yes	242	23 (9.5)	1			
	No	109	16 (14.7)	1.6 (0.83–3.24)	0.156	2.3 (0.58–8.89)	0.243
Cotrimoxazole use							
	Yes	289	36 (12.6)	1			
	No	62	3(7.7)	0.4 (0.11–1.20)	0.096	3.7 (0.98–14.35)	0.054
ARV							
	Yes	305	32 (10.5)	1			
	No	46	7 (15.2)	1.5 (0.63–3.71)	0.345	-	-
Condom use							
	Yes	197	19 (9.6)	1			
	No	154	20 (13.0)	1.4 (0.72–2.72)	0.342	1.0 (0.48–2.16)	0.963
Cd4 count							
	≤350	85	13 (15.3)	1			
	>350	266	26 (9.8)	0.6 (0.29–1.23)	0.162	0.5 (0.24–1.16)	0.114
Parity							
	Para 1	60	6 (10.0)	1			
	Para2-4	236	28 (11.9)	1.2 (0.48–3.10)	0.686	-	-
	Para >4	55	5 (9.1)	0.9 (0.26–3.13)	0.869	-	-
Sexual partners per year							
	1	306	31(10.1)	1		1	
	2	45	8 (17.8)	1.9 (0.82–4.49)	0.133	2.3 (0.90–6.23)	0.082

STIs = sexually Transmitted Infections, OR = Odds Ratio, CI = Confidence Interval

### Ethical statement

The study was approved by the Department of Obstetrics and Gynecology Mulago hospital, Makerere University School of Medicine Research and Ethics Committee and Uganda National Council for Science and Technology. The participants gave written informed consent before enrollment into the study. The names and addresses of participants were not included to maintain confidentiality.

## Results

Three hundred fifty one participants were recruited into the study. The mean age of the participants was 29.4 ± 6.2years, 83% were less than 35years, 50% had attained secondary education and nearly three quarters were married. The majority (69%) had disclosed their HIV sero status to their spouses, 82% were on Cotrimoxazole prophylaxis, 70% were on antiretroviral drugs, nearly 90% had cd4 count greater than 350, almost 60% reported condom use and nearly 70% were of parity 2–4. Over 50% of the participants’ spouses were older than 35 years, and 72% had attained secondary education (Table 1). Overall prevalence of STIs was 11.1% (95% CI 7.8–14.4) and individual prevalence for TV, NG, and CT was 5.9%, 5.4% and 0.9% respectively. Among women with STIs, TV was the commonest (46.1%) organism isolated followed by NG (41%) (Table 2). In the final logistic regression model, factors independently associated with increased risk of STI were having primary or less education (OR 2.3 95% CI 1.09–4.85) and having a spouse of primary or less education (OR 3.35% CI 1.6–6.78). Being muslim (OR 0.2 95% CI 0.04–0.78) was associated with reduced risk for STI (Table 3). The model goodness of fit was stable and had a p. value of 0.97.

## Discussion

In this study, women living with HIV/AIDS who opted for IUD were assessed for STIs using the syndromic logarithm. Participants found free and at low individual risk of STIs underwent laboratory test for NG, CT and TV at the time of IUD insertion. We found a prevalence of asymptomatic STIs of 11%, which is high. Similar findings were reported by Tyndall et.al who found a NG and CT prevalence of 10% among asymptomatic women attending a family planning clinic in Kenya [23]. Additionally, Mayer et.al reported a STI prevalence of 13% among asymptomatic HIV positive persons in primary care [24]. The sensitivity of syndromic screening to detect asymptomatic infections in this HIV positive population is questionable. Thus, these infections would have gone untreated in our setting where laboratory testing for STIs is not routinely done. Syndromic management protocols have previously been shown to perform poorly when used to screen asymptomatic populations [25,26].

Our data shows that TV and NG were the most prevalent STIs. This is consistent with prevalence studies in Uganda which have shown that TV and NG are the most prevalent STIs [11,12]. However, these studies reporting STI prevalences in Uganda were addressed at other populations and age groups. Contrary to our findings, a Kenyan study among asymptomatic women attending a family planning clinic found no difference in the prevalence of NG and CT [23]. The presence of STIs among WLHA is a public health concern since STIs are an indicator of high risk sexual behavior. High risk sexual behavior among HIV positive persons in care has been reported in Uganda and elsewhere in Africa [27,28]

Our study findings show that women with low education were more likely to have an STI. Women with primary or less education have been shown to be at an increased risk of STIs [29–31]. Less educated women lack formal employment and may be completely dependent on the male sexual partner and unable to negotiate safe sex. In addition evidence shows that low income individuals are less likely to access STI preventative information and healthcare. They are also at an increased risk of using sex for economic gains and as a psychosocial coping mechanism [28,32]. In Ghana it was shown that highly educated women are more likely to practice safer sex using condoms with their spouses compared to the poorer and less educated women [33]. Individuals with higher education have the ability to access information that helps them protect their partners and themselves from STI transmission and acquisition [34]. However, our findings are in contrast to a Gabonese study that showed that highly educated women were more likely to have STIs [35].

In sub-Saharan Africa, it has been shown that the behavior of the male partner places women at risk of STI acquisition[36]. Our data shows that women who were married to less educated men were at an increased risk of STIs. Poor education leads to unemployment and subsequently poverty. Individuals with low income have been shown to have a high prevalence of STIs[37]. This could be due to high risk sexual behavior exhibited by poorly educated men. Men have been shown to be more likely to engage in concurrent sexual relationships than women [38,39]. The high rate of high risk sexual activity among poorly educated men may explain why their wives are at an increased risk of having STIs. Studies show that women who perceive their relationships to be monogamous are less likely to use condoms and this makes them susceptible to HIV and other STIs [40,41]. Women may also be pressured to engage in unprotected sex and accept cheating men if they believe that they must compete with other women in order to win over the man [42].

Our study shows that being muslim was the only factor that was associated with a reduced risk of STIs. This may be explained by social behaviors such as ablution practiced by Muslim women soon after sex. This may be protective against STI acquisition leading to the lower STI prevalence among Muslim women. The possibility of the protective effect of male partner circumcision cannot be discounted. The majority of muslim women are married to muslim men who practice pre-pubertal circumcision. However, the impact of circumcision on STI acquisition and transmission has been controversial. Circumcision has been shown to reduce the acquisition and transmission of HIV and STIs in cross-sectional and ecological studies [43,44]. Yet longitudinal data suggests that circumcision does not reduce acquisition of STIs apart from HIV [45,46]. Although CT, NG and TV are urethral infections in men [47] the foreskin presents a conducive environment for the multiplication of pathogenic microorganisms. Thus, circumcised men with STI infections may expose their spouses to a lower pathogen burden than STI-infected uncircumcised men [45]. Furthermore muslim women abstain from alcohol consumption. Alcohol is associated with high-risk sexual behaviors which place women at an increased risk for STI acquisition [48,49].

The strength of our study is that STIs were detected using the PCR which is one of the most sensitive laboratory methods for detection of genital infections. Secondly, the study sample was randomly selected. However, there are several limitations to the study. Firstly we are reporting baseline data which was collected at the beginning of a randomized controlled trial. We cannot, therefore, ascribe causality to any of the associated factors in the study. Secondly, this was a self selecting sample of women who opted for intrauterine contraception. Women who opted for intrauterine contraception in this study were in stable sexual relationships, had children and had attained secondary education hence they were generally at low risk for STI. Therefore the results of this study cannot be generalized to all WLHA attending family planning clinics in Uganda. Thirdly, the Study participants were required to recall some issues that happened in the past. Thus, it is possible that the accuracy of information we collected could have been compromised by recall bias. This bias could have been minimal given that treatment for sexually transmitted diseases and number of sexual partners is less likely to be forgotten with in a spell of one year. Fourthly, sexual behavior is a sensitive issue especially in the context of HIV. It is therefore possible in a face to face interview that social desirability bias may have had some influences on our findings leading to under reporting of casual sexual partners and treatment for STIs. Finally several of our variables were assessed in binary fashion. This could have failed to completely eliminate confounding.

## Conclusion

The prevalence of asymptomatic STI among women living with HIV undergoing IUD insertion of 11.1% TV and NG are the commonest STIs in this population. Participants with primary or less education and those whose partners had primary or less education had a high risk while being of muslim faith was associated with a reduced risk for asymptomatic STI.
